# A systematic review of gender differences in the relationship between problematic smartphone use and depression

**DOI:** 10.1186/s40359-026-05229-3

**Published:** 2026-07-22

**Authors:** Neha Pirwani, Aleksandar Vujić, Attila Szabo

**Affiliations:** 1 Faculty of Education and Psychology, Institute of Health Promotion and Sport Sciences, ELTE Eötvös Loránd University, Bogdanfy 10/B, Budapest, 1117 Hungary; 2 Doctoral School of Education, Faculty of Education and Psychology, ELTE Eötvös Loránd University, Budapest, Hungary; 3 Department of Psychology and Healthcare Management, Faculty of Health and Sport Sciences, Széchenyi István University, Győr, Hungary

**Keywords:** Behavioral addiction, Biological sex, Mental health, Mobile phone, Psychology

## Abstract

**Background:**

Problematic smartphone use (PSU) has been increasingly linked to depression, but evidence on whether this association differs by gender remains inconsistent. This review synthesizes the existing evidence on gender differences in the PSU–depression association.

**Methods:**

Four academic databases (PubMed, Web of Science, Scopus, ProQuest) and one academic search engine (Google Scholar) were searched, with the original search conducted through September 2024 and an updated search completed in May 2026. The review was prospectively registered on the Open Science Framework and followed the PRISMA guidelines. Eligible studies examined gender differences in the association between PSU and depression. Quality was assessed using the Mixed Methods Appraisal Tool (MMAT).

**Results:**

Of the 16 included studies, 13 were cross-sectional and three longitudinal. Fifteen studies statistically tested and found a positive PSU–depression association. Gender patterns were divided: five studies found the association stronger in females, five found it stronger in males, and six found no significant gender difference in the qualitative synthesis. One longitudinal study provided direct evidence of bidirectionality.

**Conclusion:**

Findings on gender differences in the PSU–depression association are far from uniform, with comparable numbers of studies supporting female vulnerability, male vulnerability, and no difference. Observed gender patterns likely reflect moderating factors including usage type, age group, cultural context, and measurement heterogeneity rather than a fundamental difference in vulnerability between men and women. The predominance of cross-sectional designs and reliance on self-report instruments limit causal inference, and longitudinal and mechanistic research is needed to clarify the underlying associations.

**OSF registration:**

https://doi.org/10.17605/OSF.IO/E5SGN

**Supplementary Information:**

The online version contains supplementary material available at https://doi.org/10.1186/s40359-026-05229-3.

## Introduction

Smartphones have become deeply embedded in contemporary life, with an estimated 7.59 billion mobile network subscriptions worldwide in 2026 [1], reflecting the widespread adoption of mobile technologies. While smartphones offer considerable utility across work, social, and leisure domains, their pervasive use has given rise to growing concerns about problematic patterns of engagement [2].

To understand this phenomenon, a brief conceptual clarification is warranted. The literature employs a range of overlapping terms to describe maladaptive smartphone engagement, including problematic smartphone use (PSU), smartphone addiction (SA), excessive use and compulsive use, often interchangeably [3]. PSU is best understood as a broad dimensional construct referring to repetitive, maladaptive engagement with smartphones that results in negative psychological, social, or functional consequences [4, 5]. SA, by contrast, occupies the more severe end of this continuum, implying a behavioral addiction characterized by salience, loss of control, mood modification, tolerance, withdrawal, and relapse [3, 6]. Crucially, while all smartphone addicts may be considered problematic users, not all problematic users meet the threshold for addiction [7]. Neither construct is formally recognized as a clinical diagnosis in DSM-5 or ICD-11. Hedonic smartphone use: engagement driven by entertainment, social interaction, and pleasure-seeking rather than instrumental goals, is theoretically distinct from both PSU and SA, but is considered a primary precursor to problematic and addictive patterns of use [8]. In the present review, PSU is adopted as the primary overarching term, while SA is retained where it appears in cited literature or validated instruments, consistent with widespread usage in the field. It is particularly the hedonic use i.e. entertainment, social media, and gaming, that predicts PSU and SA [8]. Different usage patterns lead to various subtypes of PSU, with addiction representing the most severe manifestation [3, 9, 10].

PSU is associated with a range of mental health consequences, including depression and anxiety [11, 12], as well as poor sleep quality, impaired emotional regulation, and reduced well-being [13–15]. Depression, characterized by persistent low mood or loss of interest in daily activities, affects approximately 5% of the global adult population and is responsible for over 700,000 suicide deaths annually [16].

Importantly, individuals do not respond uniformly to smartphone use, and gender has emerged as a theoretically meaningful moderator of this relationship. Women tend to use smartphones predominantly for social and communicative purposes, such as social networking and instant messaging, while men are more inclined toward gaming and entertainment use [17, 18]. These divergent usage patterns carry distinct psychological consequences. Excessive social media engagement, particularly passive browsing, has been associated with heightened social comparison and Fear Of Missing Out (FOMO) [19], and FOMO and social anxiety are each interrelated with depressive symptoms [19, 20]. Women may therefore be more frequently exposed to these depressogenic processes given their greater engagement with social and communicative smartphone use. Gaming-related use, more prevalent among men, has been independently linked to depressive symptoms [21]. Furthermore, women are globally 1.5 times more likely than men to experience depression [16], with hormonal fluctuations and gendered social dynamics proposed as contributing biological and sociocultural mechanisms [22, 23]. Women’s higher levels of social engagement and social stress may also amplify vulnerability to habitual and addictive patterns of smartphone use [24].

Empirical findings on gender differences in PSU are, however, inconsistent. While Vujić and Szabo [8] and others [25, 26] report that women are more likely to develop PSU, Noel et al. [27] and Kalyani et al. [28] found higher smartphone addiction scores among males, and Gao et al. [29] found no significant gender differences. These conflicting findings are likely attributable to differences in the type of smartphone use examined, the populations studied, and the measurement instruments employed; precisely the kind of heterogeneity that a systematic review is well positioned to address. Moreover, no previous systematic review has specifically examined gender differences in the association between PSU and depression as its primary focus. This review therefore aims to consolidate the existing evidence on this topic and provide a more rigorous synthesis than has previously been available.

## Methods

### Inclusion and exclusion criteria

This systematic literature review included only articles published in English in peer-reviewed academic journals, with no restriction on publication date. The inclusion criteria covered all ages and ethnicities. To be included, articles had to specifically examine gender differences in the association between problematic smartphone use (PSU) and depression, with these differences reported in the results or discussion section of the paper.

The review excluded non-English articles, commentaries, abstracts, books or chapters, conference proceedings/abstracts, dissertations, editorials, literature reviews, meta-analyses, and methodological papers. Studies focusing on a single gender or a narrow population without gender comparison were excluded (see Table 1).

**Table 1:** Inclusion and exclusion criteria for the eligible studies

Inclusion Criteria	Exclusion Criteria
• Articles that specifically examine gender differences in the association between PSU and depression • Report these gender differences in the results or discussion section • Population of all ages and ethnicities • Published at any time • Published in the English language • Research articles in peer-reviewed journals	• Abstracts • Books (or chapters) • Conference proceedings/conference abstracts • Dissertations • Editorials, commentaries • Literature reviews, meta-analyses • Methodological papers • Studies focusing on a single gender or a narrow population without gender comparison

### Search strategy

Four academic databases (PubMed, Web of Science, Scopus, and ProQuest) and one academic search engine (Google Scholar) were searched sequentially. A study by Martín-Martín et al. [30] found that Google Scholar captured 95% of citations across 252 subjects, making it one of the most comprehensive sources [30]. Given its role as a search engine rather than a structured database, Google Scholar was used as a supplementary source to maximize recall, with advanced search features applied to improve consistency. We acknowledge that full reproducibility of Google Scholar searches cannot be guaranteed, and results from this source were therefore cross-checked against the four primary databases to minimize bias. Given its increasing inclusivity, we anticipated considerable overlap with the other four databases, leading to many duplicates. To minimize duplicates, Google Scholar was searched after completing the four primary databases.

The search strategy used three keyword clusters: smartphones, gender, and depression, as summarized in Table 2. Boolean logic commands were applied to refine and optimize the search. In addition to wildcard truncation, two-word phrases were also searched using quotation marks (e.g., “mobile phone”) to capture studies that may have been missed by single-term searches. This was applied alongside the standard Boolean search to maximize coverage, as different databases handle phrase searching differently.

**Table 2:** Search terms and Boolean operators used in the review

	AND		AND	
Smartphone* OR		Gender OR		Depression
Mobile phone*		Sex* OR		
		Female* OR		
		Male* OR		
		Men OR		
		Women OR		
		Girl* OR		
		Boy*		

The wildcard (*) extends the search to any ending of the word root

The review protocol was prospectively registered on the Open Science Framework (OSF) on September 13, 2024, prior to data collection (doi:https://doi.org/10.17605/OSF.IO/E5SGN), licensed under CC-By Attribution 4.0 International. No deviations from the registered protocol occurred. The pre-registration is licensed under a CC-By Attribution 4.0 International, allowing open access and reuse. This review follows the Preferred Reporting Items for Systematic Reviews and Meta-Analyses (PRISMA) guidelines [31].

### Search outcome

The original search was conducted until September 30, 2024. To capture studies published during the peer-review process, the search strategy was repeated in May, 2026, limited to records published between January 1, 2024, and May 27, 2026. This updated search was conducted across PubMed, Web of Science, Scopus, and Google Scholar using Publish or Perish software [32]. As ProQuest cannot be accessed through Publish or Perish, it was searched independently during the updated literature search. It should be noted that Publish or Perish imposes a retrieval cap of 200 records per database search and 100 records for Google Scholar; results should be interpreted with this limitation in mind.

**Fig. 1:**
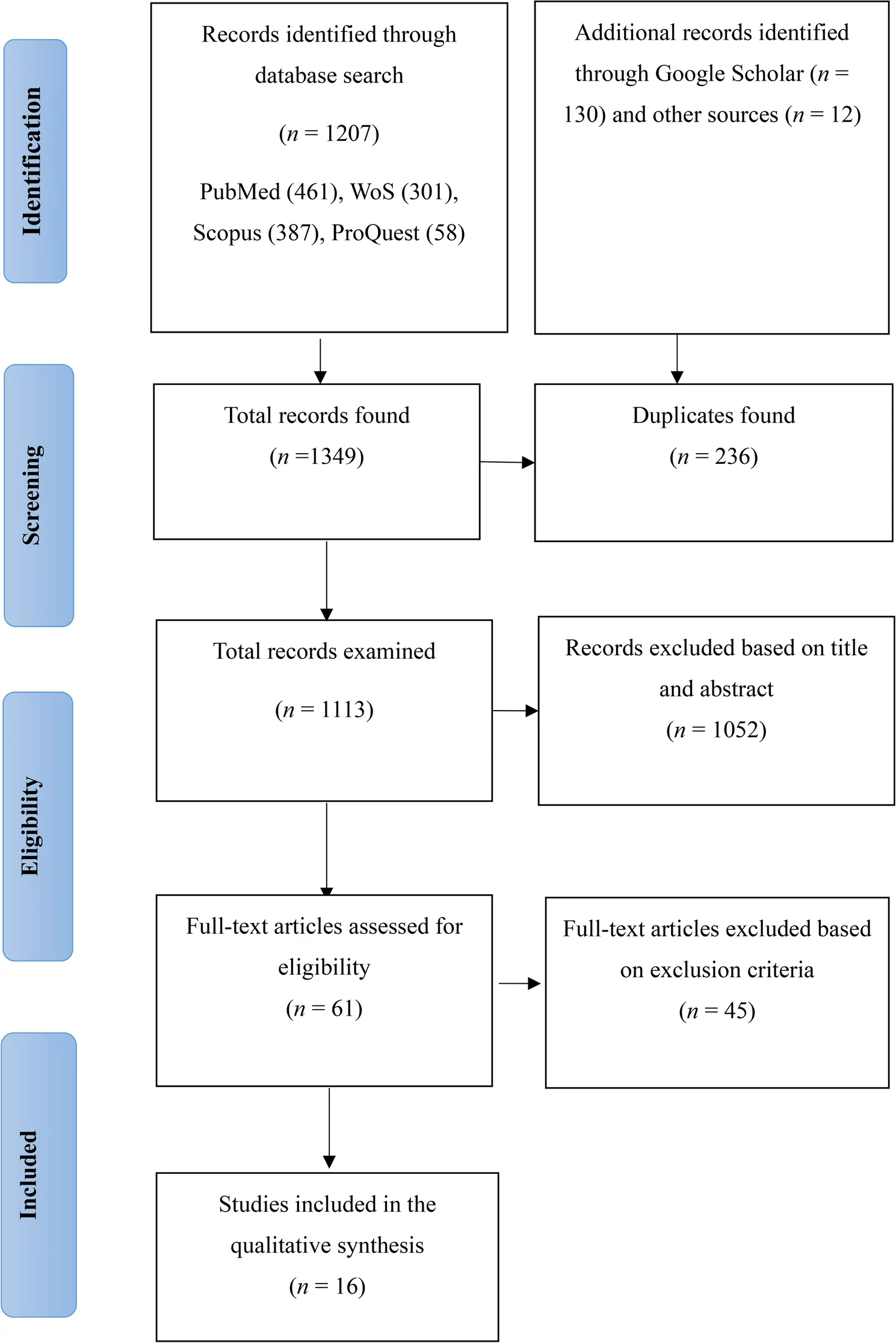
PRISMA flow diagram of the study selection process

A total of 1,207 records were identified through database searches (PubMed: 461, Web of Science: 301, Scopus: 387, ProQuest: 58), with an additional 130 records identified through Google Scholar and 12 through reference list screening, yielding 1,349 records in total. After removing 236 duplicates, 1,113 records were screened by title and abstract, of which 1,052 were excluded. Sixty-one full-text articles were assessed for eligibility, and 45 were excluded: studies were excluded if they assessed general smartphone use rather than PSU, reported gender differences in PSU and depression separately rather than examining gender differences in their association, or reported mental health outcomes too broadly for depression-specific findings to be extracted. Ultimately, 16 studies met the inclusion criteria and were included in the qualitative synthesis (Fig. 1).

Regarding study selection, screening in the original search (2024) was conducted by the first author, and screening of the updated search (2026) was conducted by the second author. Data extraction and quality assessment were performed independently and cross-checked through consensus discussion among all authors. Formal inter-rater agreement statistics were not calculated. However, consistent with MMAT guidance, quality appraisal was conducted independently by all authors, with discrepancies resolved through discussion to achieve consensus [33]. We acknowledge the absence of formal agreement statistics as a methodological limitation.

Meta-analytic pooling was considered but deemed inappropriate due to substantial heterogeneity across included studies in PSU measurement instruments, depression assessment tools, study populations, and analytical approaches. A qualitative synthesis was therefore conducted.

### Quality assessment

Three researchers independently assessed each study using the Mixed Methods Appraisal Tool (MMAT), and discrepancies were resolved through discussion until consensus was reached, consistent with MMAT guidance [33, 34]. MMAT is a widely recommended, user-friendly instrument that allows researchers to assess the quality of methodologies in qualitative, quantitative, and mixed methods studies included in systematic reviews or meta-analyses [33]. The process starts with two screening questions that determine the clarity of the research, followed by five design-specific questions. Each guiding question was evaluated as either meeting the quality standards (“Yes”), not meeting them (“No”), or being unclear (“Can’t tell”) [34].

When disagreements arose, the researchers consulted a colleague experienced in systematic reviews. This was necessary for only one study i.e. Kalyani et al. [28], in which a Chi-square independence test appeared to have been used but was not explicitly stated, and critical statistical information including p-values for the variables in question were missing. Consequently, their MMAT response for Q5 was categorized as “No” (see Table 3). Given this quality concern and the descriptive nature of their reported findings, results from Kalyani et al. [28] should be interpreted with caution. The majority of studies scored “No” on Q2 (sample representativeness), which is expected given the predominance of convenience sampling in this literature. All studies had straightforward research questions and sufficient data to address them.

**Table 3:** Quality assessment of the studies included based Mixed Methods Appraisal Tools (MMAT)

	S1-Are there clear research questions?	S2-Do the collected data allow for the research questions to be addressed?	Q1-Is the sampling strategy relevant to address the research question?	Q2-Is the sample representative of the target population?	Q3-Are the measurements appropriate?	Q4-Is the risk of nonresponse bias low?		Q5-Is the statistical analysis appropriate to answer the research question?
Ban & Kim (2024) [35]	Yes	Yes	Yes	Yes	Can’t tell	Yes	Yes	
Chen et al. (2017) [18]	Yes	Yes	Yes	Can’t tell	Yes	Yes	Yes	
Cheng et al. 2024 [36]	Yes	Yes	Yes	No	Yes	Can’t tell	Yes	
Dong et al. (2025) [37]	Yes	Yes	Yes	No	Yes	Can’t tell	Yes	
Elamin et al. 2024 [38]	Yes	Yes	Yes	No	Yes	No	Yes	
Gao et al. (2024) [29]	Yes	Yes	Yes	No	Yes	Yes	Yes	
Kalyani et al. (2019) [28]	Yes	Yes	Yes	No	Yes	Can’t tell	No	
Li et al. (2025)* [39]	Yes	Yes	Yes	No	Yes	Yes	Yes	
Liu et al. (2025)* [40]	Yes	Yes	Yes	No	Yes	No	Yes	
Nkwo et al. 2025 [41]	Yes	Yes	Yes	No	Yes	Can’t tell	Yes	
Noel et al. (2023) [27]	Yes	Yes	Yes	No	Yes	Can’t tell	Yes	
Ong et al. 2024 [42]	Yes	Yes	Yes	Yes	Yes	No	Yes	
Pan et al. (2025) [43]	Yes	Yes	Yes	No	Yes	Can’t tell	Yes	
Park & Lee (2022) [44]	Yes	Yes	Yes	Yes	Yes	Yes	Yes	
Wang et al. (2024) [45]	Yes	Yes	Yes	No	Yes	Yes	Yes	
Wang, Ma, & Meng* (2024) [46]	Yes	Yes	Yes	No	Yes	No	Yes	

*S *Screening question, *Q *Research specific questions

*longitudinal study

Once quality assessment was complete, data were extracted systematically and organized in Table 4, covering authors, publication dates, study locations, participant demographics, problematic smartphone use and depression measures, statistical tests, and key findings.

**Table 4:** Characteristics of the studies included

Author & year	Country	Study Type	Sample	Measurement	Tests and effects	Main findings
1. Ban & Kim (2024) [35]	South Korea	CS	2,404 middle-schoolers M = 1,303 boys F = 1,101 girls	Depression scale; smartphone dependency scale; aggression; cyberbullying perpetration	SEM; bootstrapping; multigroup analysis by gender	Depression predicted smartphone dependency, and this path was significantly stronger in boys than girls. Smartphone dependency fully mediated the association between depression and cyberbullying perpetration.
2. Chen et al. (2017) [18]	China	CS	1,441 Undergraduate students M = 696 F = 745 M_age_ = 19.72 ± 1.43 years	SAS-SV CES-D	Chi-square tests Multivariate logistic analysis	Depression was significantly associated with smartphone addiction in females (aOR = 1.84, 95% CI: 1.21–2.79) but not in males.
3. Cheng et al. 2024 [36]	China	CS	633 college students M = 342 F = 291 M_age_ (M) = 21.30 ± 0.28 Mage (F) = 22.30; SD ± 0.27	SAS-SV DASS-D	Correlation SEM	Depression was positively correlated with smartphone addiction (*r* = .42, *p* < .001). However, in SEM analyses separating anxiety, stress, and depression, depression did not significantly predict smartphone addiction; stress was the only significant mental-health predictor. Female students reported higher smartphone addiction, but no significant depression–smartphone addiction pathway was found in either gender.
4. Dong et al. (2025) [37]	China	CS	2,121 undergraduate students M = 981 F = 1140 M_age_ = 19.74 ± 1.82 (range 16–22)	MPATS CES-D	Mann-Whitney U Partial correlation Regression Moderated mediation (PROCESS Model 59)	Mobile phone addiction tendency positively predicted depression (β = 0.435, *p* < .001), and gender moderated this relationship (b = 0.309, ΔR² = 0.018, *p* = .010), with the association apparently stronger among female students.
5. Elamin et al. 2024 [38]	Saudi Arabia	CS	235 college students M = 78 (33.2%) F = 157 (66.8%) M_age_ = 21.5 ± 2.3	SAS-SV DASS-21 (depression)	Chi-square test Fisher exact test Logistic regression ANOVA *t*-test	PSU was more prevalent among female than male students (76.2% vs. 62.8%, *p* = .031). Depression level was significantly associated with PSU among female students (*p* = .001), but not among male students (*p* = .901). Mean smartphone addiction scores increased significantly with depression severity in the total sample (*p* < .001).
6. Gao et al. (2024) [29]	China	CS	914 adolescent middle school students M = 52.3% F = 47.7% M_age_ = 13.83 ± 0.59 (range: 12.5–16.5)	SAS-SV PHQ-9	Correlation SEM Wald and chi-square difference test	Depressive symptoms were positively associated with PSU overall (β = 0.29, *p* < .001), and in both boys (β = 0.28, *p* < .001) and girls (β = 0.32, *p* < .001). Although the overall mediation model differed by gender, Wald tests did not show a significant gender difference in the depressive symptoms to PSU path.
7. Kalyani et al. (2019) [28]	India	CS	358 Undergraduate students M = 142 F = 216 M_age_ = 19.72 ± 1.43	SAS-SV DASS-21	Prevalence comparisons (percentages)	Among those with smartphone addiction, a greater proportion of males (66%) experienced depression than females (58%).
8. Li et al. (2025) [39]	China	Longitudinal	737 middle school students M = 384 F = 353 M_age_ = 13.25 ± 0.76	MPAS PHQ-9	SEM	Depression at T2 positively predicted mobile phone addiction at T3 in the overall model (β = 0.23, *p* < .001), and in both boys (β = 0.11, *p* < .05) and girls (β = 0.21, *p* < .001). The indirect pathway through depression was significant among girls, but not boys, suggesting that depression was a stronger explanatory pathway linking bullying victimization to later mobile phone addiction among girls.
9. Liu et al. (2025) [40]	China	Longitudinal	397 college students M = 125 F = 272 M_age_ = 21.45 ± 2.21	MPAI DASS-D	Correlation SEM	Depression at T1 was positively correlated with smartphone use severity at T3 (*r* = .29, *p* < .01), but the direct depression to smartphone use path was not significant in the longitudinal SEM (β = 0.08, *p* = .20). Depression predicted later smartphone use indirectly through rumination (β = 0.15, *p* < .001). No significant sex difference was found for the depression to smartphone use pathway.
10. Nkwo et al. 2025 [41]	Predominately Nigeria, Tanzania, Ghana, and South Africa	CS	751 adults M = 304 F = 447 Age distribution: 18–20 137 18.2% 21–30 316 42.1% 31–40 196 26.1% 41–50 0 0.0% 51–60 0 0.0% > 60 102 13.6%	SAS DASS-21 (depression subscale)	PLS-SEM	Depression was positively associated with problematic mobile phone use in the overall PLS-SEM model (β = 0.25, *p* < .001). Gender-based models showed nearly identical positive associations among men (β = 0.24, *p* < .001) and women (β = 0.25, *p* < .001), indicating no meaningful gender difference in the depression–problematic mobile phone use relationship.
11. Noel et al. (2023) [27]	United States	CS	1,021 Individuals M = 132 F = 456 Other = 433 M_age_ = 21.3 ± 2.1	SAS-SV CES-DS	Univariable logistic regression models Multivariable logistic regression models	Individuals with smartphone addiction are about 2.7 times more likely to experience depression than those without it. When accounting for gender and sexual identity, heterosexual cis-males showed the strongest association between smartphone addiction and depression, compared to heterosexual cis-females and sexual/gender minorities. In this group, the likelihood of experiencing depression was 8.5 times higher for individuals with smartphone addiction than those without.
12. Ong et al. 2024 [42]	Singapore	CS	400 adults M = 200 F = 200 Age distribution: Mdn age = 42 21–30 years 105 (26.3%) 31–40 years 87 (21.8%) 41–50 years 92 (23.0%) 51–60 years 116 (29.0%)	SAS-SV PHQ-9	Logistic regression	Mild-to-severe depressive symptoms were associated with higher odds of PSU in the overall adjusted model (OR = 2.84, 95% CI [1.61, 5.02], *p* < .001). Gender-stratified analyses showed significant associations in both men (OR = 4.23, 95% CI [1.72, 10.44], *p* = .002) and women (OR = 2.15, 95% CI [1.03, 4.49], *p* = .041), with a larger OR among men.
13. Pan et al. (2025) [43]	China	CS	509 college students M = 203 F = 306 M_age_ = 19.88 ± 1.24	MPATS PHQ-9	Correlation Moderated mediation	Depression was positively associated with mobile phone addiction (*r* = .574, *p* < .01) and positively predicted mobile phone addiction (β = 0.58, *p* < .001). Gender moderated the depression to mobile phone addiction relationship (β = 0.16, *p* < .05), with simple slopes indicating a stronger association among boys (b = 0.56, *p* < .001) than girls (b = 0.40, *p* < .001).
14. Park & Lee (2022) [44]	South Korea	CS	2,056 High-schoolers M = 1041 F = 1015 M_age_ = 18	SCL-90-R SAS	Chi-square test *t*-test Multi-group path analysis	Smartphone addiction was positively associated with depression at comparable magnitudes in both male (β = 0.180) and female (β = 0.181) adolescents. However, females exhibited an additional indirect pathway from SA to depression via reduced self-control, which was not significant in males.
15. Wang et al. (2024) [45]	China	CS	7,617 college students M = 2312 (30.4%) F = 5305 (69.6%) M_age_ = 18.9 ± 0.84	MPATS PHQ-9	Chi-square test Multivariate logistic regression Odds ratio	PMPU was strongly associated with depression symptoms in the adjusted model (OR = 6.09, 95% CI [5.39, 6.88], *p* < .001). Although girls showed higher prevalence of both PMPU and depression symptoms, sex-stratified analyses found no significant sex difference in the PMPU–depression association.
16. Wang, Ma, & Meng (2024) [46]	China	Longitudinal	4,274 college students M = 20.6% F = 79.4% No age data reported (grade only: 18% freshman, 38.1% sophomore, 28.7% junior, 15.3% senior) Two-wave, 6-month interval	MPATS SDS (Zung)	Correlation Two-wave cross-lagged SEM Longitudinal measurement invariance Multi-group SEM by gender + Wald test of parameter constraints Bootstrap mediation	Smartphone addiction and depression showed reciprocal positive longitudinal associations: smartphone addiction at T1 predicted depression at T2 (β = 0.09, *p* < .001), and depression at T1 predicted smartphone addiction at T2 (β = 0.08, *p* < .001). Multi-group SEM indicated no significant gender differences in the cross-lagged paths between smartphone addiction and depression. Gender differences were found only in the autoregressive paths for depression and smartphone addiction, with stronger stability among females.

*MPATS *Mobile Phone Addiction Tendency Scale, *SAS *Smartphone Addiction Scale, *SAS-SV *Smartphone Addiction Scale–Short Version, *MPAI *Mobile Phone Addiction Index, *MPAS *Mobile Phone Addiction Scale, *SDS *Self-Rating Depression Scale [47], *PHQ-9 *Patient Health Questionnaire-9, *SCL-90-R *Symptom Checklist-90-Revised, *CES-DS *Center for Epidemiologic Studies Depression Scale, *DASS-21/D *Depression, Anxiety and Stress Scale-21 / depression subscale, *PMPU *Problematic Mobile Phone Use, *PSU *Problematic Smartphone Use, *SEM *Structural Equation Modeling, *CI *Confidence Interval, *OR *Odds Ratio, *aOR *adjusted Odds Ratio, *Mdn *Median

## Results and Discussion

Among the 16 studies included, 13 were cross-sectional and three employed longitudinal designs [39, 40, 46]. Studies were geographically diverse, with nine conducted in China, two in South Korea, and one each in Saudi Arabia, India, Singapore, the United States, and sub-Saharan Africa. Sample sizes ranged from 235 [38] to 7,617 [45] in cross-sectional studies, and from 397 [40] to 4,274 [46] in longitudinal studies. Participants spanned middle school students, high school students, college students, and general adult populations.

PSU was assessed using six instruments, most commonly the SAS-SV (seven studies) and MPATS (four studies). Depression was assessed using six instruments, most commonly the PHQ-9 and DASS-21 (see Table 4). This variability introduces heterogeneity that limits direct cross-study comparability of effect sizes. Where studies reported different statistics (β, OR, r), these are reported as available; formal pooling was not possible. Fifteen of the 16 included studies statistically tested and found a positive association between PSU and depression. Kalyani et al. [28] reported descriptive prevalence data consistent with this direction but did not test the association statistically. Findings are organized below by gender pattern.

### Female-predominant pattern

Four cross-sectional studies and one longitudinal study [35; discussed below] found the PSU–depression association to be stronger in or exclusive to females. Chen et al. [18] found depression significantly associated with smartphone addiction among females (aOR = 1.84, 95% CI: 1.21–2.79) but not males. Elamin et al. [38] similarly found depression severity associated with PSU among females (*p* = .001) but not males (*p* = .901). Dong et al. [37] found gender moderated the PSU–depression relationship (b = 0.309, ΔR² = 0.018, *p* = .010), with a stronger association among females. Park and Lee [44] found comparable direct associations in males (β = 0.180) and females (β = 0.181), but females exhibited an additional indirect pathway through reduced self-control, absent in males. This suggests that while the direct PSU–depression link may be similar across genders, females may be additionally vulnerable through secondary psychological mechanisms.

### Male-predominant pattern

Five cross-sectional studies found the association stronger in males. Ban and Kim [35] found the depression to smartphone dependency path approximately three times stronger in boys (B = 0.308, *p* < .001) than girls (B = 0.097), with a significant gender difference (critical ratio = − 2.830, *p* < .01). The authors contextualized these findings within theories of masculine emotional socialization [48], proposing that adolescent boys may be less likely to externalize or openly communicate depressive symptoms, potentially increasing their vulnerability to maladaptive coping strategies such as PSU. Noel et al. [27] found that while smartphone addiction was associated with 2.7 times greater odds of depression overall, heterosexual cisgender males showed the strongest association (OR = 8.45), though this should be interpreted cautiously given the small male subsample (*n* = 132). Ong et al. [42] found significant associations in both men (OR = 4.23) and women (OR = 2.15), with a notably larger effect in men. Pan et al. [43] found gender moderated the depression to PSU relationship (β = 0.16, *p* < .05), with stronger slopes in males (b = 0.56) than females (b = 0.40). Kalyani et al. [28] found a higher proportion of males (66%) than females (58%) experienced depression among those with smartphone addiction, though this study relied on descriptive percentages only and should be interpreted with caution given its MMAT quality concerns.

### No significant gender difference

Four cross-sectional and two longitudinal studies [36, 37; discussed below] found no significant gender difference in the PSU–depression association. Gao et al. [29] found comparable associations in boys (β = 0.28) and girls (β = 0.32), with Wald tests confirming no significant gender difference. Wang et al. [45] found a strong overall association (OR = 6.09, 95% CI [5.39, 6.88]), but sex-stratified analyses found no significant difference despite girls showing higher prevalence of both PSU and depression. Nkwo et al. [41] found nearly identical associations in men (β = 0.24) and women (β = 0.25) in a sub-Saharan African adult sample. Cheng et al. [36] found a positive overall correlation (*r* = .42, *p* < .001), but when anxiety and stress were modeled simultaneously, depression did not independently predict PSU in either gender; stress was the only significant mental health predictor. This suggests that depression’s apparent association with PSU may be partially confounded by co-occurring stress and anxiety, a methodologically important finding that warrants attention in future research.

These null-difference findings are not trivial; they suggest that the PSU–depression association may be robust across genders in certain populations and contexts, and that gender differences, where found, may reflect moderating variables such as usage type, age group, or cultural context rather than a fundamental difference in vulnerability.

### Longitudinal evidence and bidirectionality

Three longitudinal studies addressed directionality. Wang, Ma, and Meng [46] conducted a two-wave cross-lagged SEM study over six months (*N* = 4,274), finding bidirectional associations: PSU predicted later depression (β = 0.09, *p* < .001) and depression predicted later PSU (β = 0.08, *p* < .001), with no significant gender differences in these paths. Li et al. [39] found depression predicted later mobile phone addiction in both boys (β = 0.11, *p* < .05) and girls (β = 0.21, *p* < .001), with the indirect pathway through depression significant among girls but not boys. Liu et al. [40] found no significant direct longitudinal path from depression to PSU (β = 0.08, *p* = .20), but an indirect path through rumination (β = 0.15, *p* < .001); no gender difference was found in the depression to PSU pathway. This highlights rumination as a potential psychological mechanism linking depression to PSU and underscores the importance of examining indirect pathways rather than direct effects alone.

Of the three longitudinal studies, only Wang, Ma, and Meng [46] tested both directions and provided direct evidence of bidirectionality. The other two studies [39, 40] examined only the depression to PSU direction, both finding significant associations either directly or through indirect pathways. While this limited longitudinal evidence suggests that depression can predict later PSU and may be reciprocally related to it, more longitudinal studies testing both directions are needed before a bidirectional relationship can be confidently established. If supported by future research, this would have important clinical implications, as PSU and depression may reinforce each other over time, and intervention targeting either construct may benefit the other.

### Sociocultural and psychological explanations

Gender differences across studies are unlikely to reflect a single mechanism. Excessive social media engagement, particularly passive browsing, has been linked to social comparison, FOMO, and depressive symptoms [19]. Women’s greater engagement with social and communicative use may therefore expose them more frequently to these depressogenic processes [49]. Hedonic social use has been identified as a primary precursor to PSU [8], and women’s greater engagement with this pattern may partly explain their elevated vulnerability. For men, gaming-related smartphone use may represent a distinct pathway. Internet gaming is more prevalent among males and consistently associated with depressive symptoms [21]. Additionally, masculine emotional socialization: the cultural expectation that men suppress rather than express emotional distress [48], has been proposed as a mechanism through which male adolescents redirect depressive affect into smartphone dependency rather than expressing it directly [35]. This may suggest that male depression manifests through behavioral avoidance, including smartphone overuse, rather than direct affective expression. Consistent with escape theory [50], males experiencing depression may turn to immersive smartphone activities such as gaming as a means of escaping negative affect, a mechanism that may partly explain the male-predominant patterns observed in several included studies. Biological explanations, including hormonal contributions to women’s higher global depression rates [22], are plausible but should be treated cautiously here, as no included study examined hormonal mechanisms directly.

### Limitations

Several limitations should be acknowledged. First, considerable heterogeneity in measurement instruments was observed, with PSU and depression each assessed using six distinct tools across included studies. This variability, combined with inconsistent reporting of effect sizes (β, OR, r, or descriptive percentages), limits cross-study comparability and precluded formal meta-analytic pooling. Second, all included studies relied on self-report measures of both PSU and depression. Shared method variance may inflate observed associations, as depressed individuals may be more likely to perceive their smartphone use as problematic regardless of actual usage. Future research should incorporate objective measures such as screen time tracking. Third, the predominance of cross-sectional designs limits causal inference. Only three longitudinal studies were included, and PSU and depression may be reciprocally related rather than unidirectionally linked. Plausible mediating mechanisms including poor sleep, emotional dysregulation, and reduced well-being have been associated with both constructs [13, 51–53], but their causal role remains unclear. Relatedly, evidence for bidirectionality between PSU and depression is currently limited to a single longitudinal study [46] and should be interpreted as preliminary rather than conclusive. Fourth, restriction to English-language publications may have introduced language bias, particularly given the substantial body of PSU research published in Asian languages. Although nine included studies were conducted in China and two in South Korea, relevant non-English studies may have been excluded. Fifth, cultural variability in smartphone use norms, gender role expectations, and depression expression across the diverse contexts represented (East Asia, South Asia, Middle East, North America, sub-Saharan Africa) may influence findings in ways this review did not formally examine. Sixth, most included studies sampled adolescents and university students, with only three including general adult populations. Findings may therefore have limited generalizability to older adult populations. Finally, publication bias toward positive findings cannot be discounted, as studies reporting significant or gender-differentiated associations may be preferentially published over null findings, potentially inflating the apparent strength of this relationship.

### Future implications

Future research should prioritize longitudinal and experimental designs to clarify the directionality of the PSU–depression relationship across genders, including potential mediating mechanisms such as rumination, sleep disturbance, and emotional regulation. Cross-cultural studies are needed to determine whether observed gender differences generalize beyond the contexts represented in current literature, and research extending beyond adolescent and student samples is essential for broader public health relevance. Methodologically, future studies should incorporate objective smartphone use measures. Examining specific usage patterns i.e. social networking, gaming, passive browsing, active communication as distinct predictors would clarify the mechanisms underlying gender differences. Gender-sensitive interventions may be most effective in addressing the PSU–depression relationship, complemented by broader measures including digital literacy education, mental health awareness programs, and mindfulness-based approaches.

## Conclusion

This systematic review of 16 studies confirms a consistent positive association between PSU and depression, but the gender patterning of this association is far from uniform. Five studies found the association stronger in females, five found it stronger in males, and six found no significant gender difference. This balanced distribution suggests that gender differences in the PSU–depression relationship are not as definitive as the broader literature has implied, and likely reflect moderating factors such as usage type, age, and cultural context rather than a fundamental difference in vulnerability between men and women. Emerging longitudinal evidence further complicates a simple gendered narrative: PSU and depression may influence each other over time, though current evidence on bidirectionality rests on a single longitudinal study and warrants further confirmation. Future research should prioritize longitudinal and mechanistic designs to clarify the underlying pathways, particularly the role of mediators such as rumination, sleep, and emotional regulation. From an intervention standpoint, gender-sensitive strategies addressing usage-specific patterns, for instance, social comparison and FOMO associated with social networking, or escape-driven engagement associated with gaming may be more effective than uniform population-level approaches.

## Supplementary Information

Supplementary Material 1.

Supplementary Material 2.

## Data Availability

No datasets were generated or analysed during the current study.

## Abbreviations

PSU: Problematic Smartphone Use
SA: Smartphone Addiction
FOMO: Fear of Missing Out
DSM: 5–Diagnostic and Statistical Manual of Mental Disorders, 5th Edition
ICD: 11–International Classification of Diseases, 11th Revision
PRISMA: Preferred Reporting Items for Systematic Reviews and Meta–Analyses
OSF: Open Science Framework
MMAT: Mixed Methods Appraisal Tool
OR: Odds Ratio
aOR: Adjusted Odds Ratio
CI: Confidence Interval
SEM: Structural Equation Modeling
